# The use of information technology to improve interdisciplinary communication during infectious diseases ward rounds on the paediatric intensive care unit

**DOI:** 10.1038/s41598-024-51986-9

**Published:** 2024-01-18

**Authors:** Jef Willems, Adeline Heyndrickx, Petra Schelstraete, Bram Gadeyne, Pieter De Cock, Stien Vandendriessche, Pieter Depuydt

**Affiliations:** 1 Department of Critical Care, Paediatric Intensive Care Unit, Ghent University Hospital, 1K12-D, Corneel Heymanslaan 10, 9000 Ghent, Belgium; 2 Department of Paediatrics, Ghent University Hospital, Ghent, Belgium; 3 Department of Paediatrics, Paediatric Pneumology and Infectious Diseases, Ghent University Hospital, Ghent, Belgium; 4 Department of Critical Care, Intensive Care Unit, Ghent University Hospital, Ghent, Belgium; 5 Department of Pharmacy, Ghent University Hospital, Ghent, Belgium; 6 Department of Basic and Applied Medical Sciences, Ghent University, Ghent, Belgium; 7 Department of Laboratory Medicine, Medical Microbiology, Ghent University Hospital, Ghent, Belgium

**Keywords:** Health care, Medical research

## Abstract

Prospective audit with feedback during infectious diseases ward rounds (IDWR) is a common antimicrobial stewardship (AMS) practice on the Paediatric Intensive Care Unit (PICU). These interdisciplinary meetings rely on the quality of handover, with high risk of omission of information. We developed an electronic platform integrating infection-related patient data (COSARAPed). In the mixed PICU of a Belgian tertiary hospital we conducted an observational prospective cohort study comparing patient handovers during IDWRs using the COSARAPed-platform to those with access only to conventional resources. The quality of handover was investigated directly by assessment if the narrative was in accordance with Situation-Background-Assessment-Recommendation principles and if adequate demonstration of diagnostic information occurred, and also indirectly by registration if this was only achieved after intervention by the non-presenting AMS team members. We also recorded all AMS-recommendations. During a 6-month study period, 24 IDWRs and 82 patient presentations were assessed. We could only find a statistically significant advantage in favor of COSARAPed by indirect evaluation. We registered 92 AMS-recommendations, mainly resulting in reduced antibiotic pressure. We concluded that the IDWR is an appropriate platform for AMS on the PICU and that the utilisation of COSARAPed may enhance the quality of patient handover.

## Introduction

Antibiotic resistance has long been recognised as a major health threat and usage of (broad spectrum) antibiotics as a key driver in its development^1^. Antimicrobial stewardship (AMS) promotes responsible antibiotic usage, with the goal to preserve the effectiveness of the drugs and to protect our patients from unintended consequences of antimicrobial use^2^.

While the prevalence of infection and sepsis is high on the Paediatric intensive care unit (PICU), with an estimated 40-80% of patients receiving antimicrobial drugs^3^, inappropriate prescribing (mainly overly broad spectrum and wrong dosage) ranges up to 60%^4^.

Prospective audit with feedback (PAWF), conducted by a so-called AMS team has been demonstrated to result in a significant reduction in antimicrobial usage in children’s hospitals^5^, usually by performing antibiotic de-escalation (ADE). ADE can be achieved by replacing one antibiotic by another with narrower spectrum, by reducing the number of antibiotics in case of combination therapy or by discontinuation of antimicrobial therapy. Though widely applied, effects on antimicrobial resistance have not been demonstrated so far^6,7^. To compare and to rank the spectrum of antimicrobial drugs is subject to debate, with consensual agreement for beta-lactam antibiotics being available^8^.

The infectious diseases ward round (IDWR) is the classic setting for PAWF, with proven effectiveness in reducing antibiotic consumption in the PICU^9^. Though the interdisciplinary communication surrounding sharing of care has been identified as a crucial domain for quality improvement by governing bodies^10^, few studies aiming at improvement of patient handover, usually focusing on the daily (bedside) interdisciplinary (medical and nursing) ward rounds, have been conducted^11^. Interventions usually involve introduction of checklists and implementation of standardised handover protocols. The communication tool SBAR (situation-background-assessment-recommendation) has been developed to increase handover quality and is commonly used. However high-quality research on its ability to improve patient safety is lacking^12^. Information Technology (IT) has massively gained ground on the data-intense environment of the (P)ICU during the last two decades^13^. Classic IT-models include monitoring and data acquisition systems, integrating them in an electronic medical and nursing record. Automated drug and test order systems may reduce prescription errors^14^. Clinical decision support systems (CDSS) may help to triage patients in Emergency Departments, or detect deteriorating paediatric patients on general wards by using paediatric early warning systems (PEWS)^15,16^. Also in the field of AMS, IT has gained ground, amongst others by integrating surveillance data in clinical care electronic systems^17,18^. The use of computerised rounding systems has shown to improve quality and time-efficacy of resident rounds^19,20^. In this study, we firstly qualitatively assessed the handover during the interdisciplinary IDWR, hypothesising that quality and time-efficacy would benefit from the usage of IT. Secondly, we documented IDWR recommendations and their implementation rate.

## Methods

### Setting and study design

A prospective observational study was conducted on the PICU of the University Hospital Ghent, a tertiary teaching hospital, between November 12th 2019 and July 28th 2020. The PICU is a 14-bed, mixed medical-surgical-cardiac surgical unit to which approximately 1000 critically ill children are admitted annually. The IDWR, in which all admitted patients with infectious problems or with antibiotic prescriptions are discussed on a weekly basis in a separate staff meeting room, is attended by the AMS-team: a PICU-consultant, a paediatric infectious diseases (PID) consultant, a senior clinical pharmacologist and a senior microbiologist. First the junior PICU-staff presents the patients history with focus on infectious problems and antimicrobial management and projects ‘crucial diagnostic resources’ as microbiology and laboratory results and radiologic findings on a wall screen. Subsequently the AMS team formulates their recommendations.

Junior PICU staff, both paediatricians and emergency physicians in training, rotate per 3 to 6 months. During their first introduction to the PICU, they receive guidance both on how to conduct patient handover according to SBAR principles and on which resources to present during the IDWR.

The COSARA (Computer-based Surveillance and Alerting of nosocomial infections, Antimicrobial Resistance and Antibiotic consumption in the ICU) -platform, developed by a consortium of the Ghent University Hospital and University Ghent (Belgium)^21^, integrates all infection-related data present in ICU by continuous real-time synchronisation of the newly created database with originating sources including the (bedside) Intensive Care Information System (including all monitoring parameters, medication prescriptions, ventilator settings, temperature measurement, observations e.g. all catheter-related procedures), picture archiving and communication systems (PACS) and general laboratory information management system (GLIMS). Also registrations by clinicians are processed, including classification of proven or presumed infections, linking of antibiotic prescriptions to microbiology results and documentation of the clinical decision making process behind each antibiotic prescription. Registration is finalised and stored after formal review and corrections by dedicated senior staff. These data are for each patient individually presented in one clear overview of all infections, antibiotic treatments and associated cultures and antibiograms, using visual bars on timelines and graphs. By clicking these bars, the clinical reasoning behind antibiotic treatment becomes available for appraisal also when the prescribing clinician is absent. X-rays, microbiology history and catheter-related details (again visualised using bars on timelines) are separately available within the same platform. COSARA has contributed to the construction of multifaceted datasets on both antibiotic use and infection diagnoses in our center^22^.

As community-acquired infections (both viral and bacterial) are relatively more common in PICU if compared to the adult ICU, in 2014 a paediatric version, COSARAPed, was constructed, with a clinical decision registration module adapted to the epidemiology of PID. During the study period, the IDWR was conducted on a weekly alternating base using either the COSARAPed platform either ‘conventional’ resources (including access to source documents from separate servers as electronic patient records, GLIMS and PACS) to present all relevant patient details.

Informed consent was obtained from all the participating team members. Ethical committee Approval by the ethical committee of Ghent University Hospital was obtained (reference number 2019/1504 BC-04098). All research was performed in accordance with relevant guidelines/regulations.

### Outcomes

For each presented patient, following variables were collected by a single observer who did not participate nor interfere during the ward round (JW): usage of the COSARAPed platform or ‘conventional resources’, attendance of staff, duration of presentation and discussion.

The quality of handover was assessed in four different ways:

1. Directly by recording if the narrative of the presentation by the presenting junior staff member was in accordance with SBAR principles (Yes(Y)/No(N)).
2. Indirectly by recording if necessary history and clinical details were only complete after interference (requests, additions, corrections) by the AMS-team (Y/N).
3. Directly by recording if all necessary source documents were spontaneously and adequately projected (Y/N).
4. Indirectly if the necessary source documents were only shown after request by the AMS-team (Y/N).

For each individual patient, the total duration of the handover and discussion was timed.

Following AMS recommendations were recorded : initiation, escalation, ADE, scheduled stop orders of antimicrobial therapy; adjustment of antibiotic dosage and planning of additional diagnostic investigations. Implementation of the recommendations made by the AMS team was registered (Y/N).

Possible clinical barriers for ADE were documented as well: septic shock as per sepsis classification, immunocompromised patient status, high risk of undiagnosed pathogens, colonisation with multi-drug resistant (MDR)-pathogens.

General outcome variables including length of stay (LOS), days of antimicrobial therapy (DOT, defined as number of days with systemic administration of at least one dose of an antimicrobial) as well as PICU-mortality were registered.

### Statistical analysis

Statistical analysis of the results was performed using Statistical Package for the Social Sciences, Version 26 (SPSS, Chicago, IL). Descriptive statistics were done for baseline demographics and clinical characteristics. Categorical data were compared using Pearson Chi^2^ Tests, and independent t-test for the age distribution. Dichotomic variables were analysed with logistic regression analysis, assessing the null-hypothesis that there was no difference between the usage of the COSARAPed platform and the usage of conventional resources. Fisher’s Exact Test was used in case of few expected observations. Continuous variables and outcomes (duration of presentation and discussion, LOS and DOT) were analysed with linear regression analysis. A *p*-value less than or equal to 0.05 was considered statistically significant for all analyses.

## Results

### Ward round characteristics (Table 1)

**Table 1:** Patient and Ward round characteristics.

	COSARAPed (N 37)	Conventional Resources (N 45)	P-value
Age	6 m (med) (4 d –17 y)	17 m (med) (8 d-15 y)	0.388
Gender	M 20/37 F 17/37	M 24/45 F 21/45	0.95
Clinical barriers for AS			
Immunodeficiency	4/37	5/45	0.97
Septic Shock	2/37	2/45	0.84
Colonisation MDR pathogens	3/37	3/45	0.80
High risk of undiagnozed pathogens	5/37	10/45	0.31
Consultant PICU present	34/37	26/45	0.001
Junior staff PICU present	37/37	45/45	1
PID consultant present	37/37	45/45	1
Microbiology consultant present	37/37	45/45	1
Clinical pharmacist present	32/37	37/45	0.59
General outcome variables			
LOS PICU (days)	5 (med) (IQR 5)	5 (med) (IQR 7)	0.75
Mortality	1/37	1/45	0.89
DOT antimicrobials (days)	9 (med) (IQR 10)	7 (med) (IQR 12)	0.34

*AS* Antibiotic stewardship, *COSARAPed* Computer-based Surveillance and Alerting of nosocomial infections, Antimicrobial Resistance and Antibiotic consumption in the ICU Pediatric, *d* days, *DOT* Days of therapy, *F* Female, *IQR* Inter-quartile range, *LOS* Length of stay, *M* Male, *m* Months, *MDR* Multi-drug resistant, *med* median, *N* Number, *PICU* Pediatric intensive care unit, *PID* Pediatric infectious diseases, *y* years.

Data of Table 1 were compared using Pearson Chi^2^ test and independent t-test for the age. LOS and DOT were analysed with multiple logistic regression analysis. A *p*-value less than or equal to 0.05 was considered statistically significant.

During 24 IDWRs, 204 individual patient cases were considered for discussion. Ninety-four patients were not discussed because they either had no infectious problems or were on prophylactic antibiotics only (most often routine peri-operative prophylaxis). We only included the first presentation and discussion for each individual patients, resulting in a further exclusion of 28 presentations and a remaining 82 analysed patients. Of these, 37 (45%) were presented using COSARAPed and 45 (55%) using conventional resources. Groups were comparable in regard of age and gender but also in terms of potential clinical barriers for AS and in terms of general outcome variables (LOS, DOT and mortality).

Staff attendance during the IDWR was complete in 18/24 (75%) meetings. Senior PICU-staff attended in 20/24 (83%) and senior clinical pharmacist in 22/24 (92%) meetings. All other team members (PID consultant, senior microbiologist and junior PICU staff) were present on all occasions (100%). Senior PICU staff attendance was significantly lower when conventional resources were used (*p*-value 0.001).

### Quality and Time-efficiency of handover during IDWR (Table 2)

**Table 2:** Quality and duration of handover.

Dichotomic outcomes	COSARAPed (N 37)	Conventional Resources (N 45)	*P*-value	OR (LL-UL)
Adequate SBAR*	36/37 (97%)	39/45 (87%)	0.12	*
Adequate Resources	25/37 (68%)	26/44 (59%)	0.23	0.54 (0.19-1.51)
Interference SBAR	6/37 (16%)	16/44(36%)	0.04	3.37 (1.03-11.05)
Interference Resources	2/37 (5%)	10/44 (23%)	0.02	11.89 (1.31-106.26)
Continuous outcomes	COSARAPed (N 37)	Conventional Resources (N 45)	*P*-value	B (LL-UL)
Duration handover	4’26” (IQR 3’42”)	4’10” (IQR 6’21”)	0.75	-17.99 (-128.21-92.23)

*COSARAPed* Computer-based Surveillance and Alerting of nosocomial infections, Antimicrobial Resistance and Antibiotic consumption in the ICU Pediatric, *IQR* Inter-quartile range, *N* Number, *SBAR* Situation-background-assessment-recommendation.

Data in Table 2 were analysed using multiple logistic regression or *Fisher’s Exact Test.

Direct assessment of the quality of the handover demonstrated a tendency towards better compliance with SBAR principles (97% vs 87%, *p*-value 0.998) and towards more complete presentation of infection-related test results (68% vs 59%, *p*-value 0.23) in the COSARAPed-guided ward rounds, albeit not significant.

Indirect assessment showed a statistically significant difference in favour of COSARAPed, with 16% versus 36% interference (*p*-value 0.04) during the patient presentation and 5% versus 22% interference (*p*-value 0.02) during the projection of the relevant data with utilisation of COSARAPed if compared to conventional resources.

No difference could be observed regarding the duration of the case-presentations/discussions in the two groups, with a median duration of 4’26”” (IQR 3’42”) in the COSARAPed group versus 4’10” (IQR 6’21”) in the conventional resources group.

### AMS interventions and outcome (Table 3)

**Table 3:** AMS recommendations and implementation.

AMS therapeutic recommendations	COSARAPed (N 37)	Conventional Resources (N 45)
Scheduled stop order	14/37	12/45
Start antibiotics	1/37	1/45
Escalation antibiotics	3/37	6/45
ADE (change to narrow spectrum)	2/37	3/45
ADE (stop antibiotic or reduce number)	5/37	9/45
Dose-optimisation	3/37	4/45
No intervention	13/37	16/45
Recommendations followed	40/41	51/51

*ADE* Antibiotic de-escalation, *AMS* Antibiotic stewardship, *COSARAPed* Computer-based Surveillance and Alerting of nosocomial infections, Antimicrobial Resistance and Antibiotic consumption in the ICU Pediatric, *N* Number.

Overall, 92 therapeutic recommendations were made in the 82 studied patients, including 63 changes to the antibiotic regimen in 53 patients. Most often these changes involved a scheduled stop date or ADE. ADE included discontinuation of antimicrobial therapy, reduction of the number of antibiotics and change to antibiotics with a more narrow spectrum.

Furthermore, in 11 patients it was advised either to start antibiotics or to escalate to a broader antibiotic spectrum. In 7 patients dose optimisation was recommended. In some patients more than 1 recommendation was made. Clinical confounders known to hamper ADE, including risk factors as colonisation with MDR-pathogens, infections with a high risk of undiagnosed pathogens (e.g. abdominal sepsis), disease severity (septic shock according to sepsis classification)^23^ and immunodeficiency were often seen (Table 1). There was no statistical difference between the two studied groups for any of these variables.

In a further 29 patients, it was advised not to change the antimicrobial therapy.

The implementation rate of the recommendations was very high in our study group, with 90/92 of therapeutic recommendations followed in clinical practice. In both cases where the recommendations were not followed, unforeseen complications had occurred.

## Discussion

Whilst evaluating the quality of handover during the IDWR, we could observe an indication of a positive impact of the use of the electronic platform COSARAPed, though our findings could not be demonstrated statistically.

PICU-patients with infectious problems represent a very complex population with an often complicated treatment history and with a vast amount of available diagnostic data. However barriers to information access exist, with the need to scroll and click through different screens to review necessary information and limits to the amounts and types of information simultaneously visible on one screen. Software often lacks user-friendliness with the time-consuming process to access different servers with need to re-enter passwords multiple times. Clinical decision making may be very complicated in critically ill patients and as such these barriers may interfere with quality of decision-making^24^.

The results of laboratory tests and imaging investigations directly impact diagnostic and therapeutical clinical decision making and incomplete follow-up of test results represents a key patient safety issue^25^. Additionally, the intention behind therapeutic decision making is often not entirely clear from the patient record (Supplementry information).

The quality of often very complex case presentations is a crucial factor for correct clinical decision making during an IDWR, where AS team members rely heavily on the transfer of information, usually done by junior medical staff. Due to changing working time directives, several junior doctors on rotation will be in charge for the same patient and continuity of care may be hampered^26^.

We evaluated not only handover omissions during the IDWR by direct assessment of the quality of the handover according to SBAR-principles and by recording if projection of relevant diagnostic resources occurred, but also potential handover omissions by recording of interference (additions, corrections, queries) by the non-presenting attending staff.

In our setting, the use of COSARAPed resulted in significantly less interference by the AS-team. We could hypothesise that in the absence of this interference, incomplete information could have been given, potentially resulting in inappropriate AS interventions and thus preventable patient harm.

This is an important finding as it suggests that the reliability of the handover may be higher when using IT as COSARAPed, especially when ward rounds are conducted by less experienced teams. Whether this improves the quality of the AS-interventions and patient outcome requires further investigation.

Senior PICU-staff attended the IDWR-discussions for significantly fewer patients when conventional resources were used. It is unclear if this has influenced our study results. However, as they often interfere during SBAR, it is possible that the difference between the two groups would have been even larger with equal attendance rates.

We hypothesised that utilization of the COSARAPed platform would be time-saving, since all relevant resources are present on one screen-presentation instead of requiring logging into different servers. We have not been able to demonstrate this. As we did not separately quantify the time required for the patient-presentation itself but also included the time spent for discussion and formulation of recommendations afterwards, the full time-saving effect on the presentations alone probably is not demonstrated.

The most challenging and complex patients are those with prolonged PICU-stay, with higher risk of hospital-acquired infections (with possibly more MDR pathogens), possibly prolonged invasive mechanical ventilation or more catheter days. These patients were discussed multiple times, however only the first encounter was registered in this study, to exclude a bias of previous knowledge by team-members or possibly careless repeat-presentations. We could hypothesise however that the positive impact of COSARAPed would be even larger in these patients.

The high number of recommendations demonstrates the value of IDWR with PAWF as an AMS intervention. The most frequent therapeutic recommendations involved a scheduled antibiotic stop date and ADE. Of note, also recommendations resulting in initiation of antibiotic treatment or in escalation to antibiotic treatment with broader spectrum were made, demonstrating the primary aim of AS: to treat efficiently where needed.

A lesson to be learned may be that in our setting, compliance with the recommendations was very high, presumably due to the frequent attendance of PICU-consultants during the IDWR. This could defer difficult clinical decisions to the scene of the IDWR, rather than afterwards at the bedside.

This study has important limitations. Junior staff received only didactic and informal training at the onset of their rotation. Simulation-based training could have resulted in better quality of presentation.

The design where data are collected through direct observation by the principal investigator may induce an observer-expectancy bias (“Hawthorne effect”). Since the use of COSARAPed was obvious, blinding of the investigator was not feasible.

Equally, an observer effect may be suspected, where participants change their behavior simply being aware to be observed and evaluated, even if they were not informed on which aspects of the communication process were analysed.

The composition of the AS-team was not constant, with different junior and senior PICU staff attending the IDWR. Individual team-members may have a different approach to multidisciplinary discussions and to AMS. Junior staff may have a learning curve when presenting patients in a qualitative way and they also rotate every 3-6 months. We tried to eliminate these biasing factors by alternating usage of COSARAPed and conventional resources on a weekly basis.

When conducting the IDWR once weekly, only about 40% of the annually admitted patients would be considered for discussion. Hypothesising that mainly short admissions are overseen and that complex cases with prolonged PICU-stay would be discussed regularly, we believe most major infectious problems are detected this way. Clearly a more frequently conducted IDWR would offer better AMS opportunities.

## Conclusions

Utilisation of COSARAPed as an electronic information and communication tool may enhance time-efficiency, standardisation and quality of patient presentation during IDWRs conducted on the PICU, contributing to AMS initiatives. It may result in a reduction of handover omissions and may allow conduction of the IDWR in a reliable way on junior staff level. Its impact on the quality of the recommendations and on patient outcome requires further study.

Non-technical skills, implementation processes and education of staff are increasingly recognised as a key research domain. As such, the interdisciplinary ward round deserves further attention. To improve not only the amount and quality of information transferred during the ward round, but also clinical outcomes, future research should feature robust study design.

### Supplementary Information

Supplementary Information.

## Data Availability

Most data analysed during this study are included in this published article and its online resources. The underlying datasets generated during the current study are available from the corresponding author on reasonable request.
