# Supplementary material for: The use of information technology to improve interdisciplinary communication during infectious diseases ward rounds on the paediatric intensive care unit
Source: Sci Rep. 2024 Jan 18;14:1657. doi: 10.1038/s41598-024-51986-9 (PMC10796760; doi:10.1038/s41598-024-51986-9)
Supplement: Supplementary file 1 — Supplementary Information. [file 41598_2024_51986_MOESM1_ESM.pdf]

Supplement 1: Infectious Diseases Ward Round Patient Presentation by Junior medical PICU-staff

PART 1: Case presentation by a junior medical staff member according to the (I)SBAR principle. The junior medical staff member projects all relevant data on the screen. Alternate use of COSARAPed and traditional individual sources (GLIMS, EPD, PACS).

**I**dentification	• name<br>• age<br>• length of stay<br><br>*Isbar, 4 years old boy, admitted 5 days ago*
**S**ituation	• reason for admission<br>• current clinical status (stable/unstable)<br>• degree of organ failure<br><br>*Polytrauma after road traffic accident. Neurotrauma (skull base fracture with cerebrospinal fluid leak and extensive subdural hematoma) for which urgent neurosurgery (decompressive bone flap en placement of and external ventricle drain) at the moment of admission.*<br><br>*Blunt thoracic and abdominal trauma handled conservatively.*<br><br>*Currently sedated and ventilated which blood pressure support in the context of neuroprotection.*<br><br>*Central venous access in place.*
**B**ackground	• infectiological history<br><br>*At day two of admission there was fever and purulent sputum for which amoxicillin/clavulanic acid was start intravenously because of suspicion of ventilator acquired pneumoniae.*
**A**ssessment	• infectiological data (cultures, inflammatory blood tests, viral tests,

	radiology)<br><br>*CRP escalated to 140 mg/L, white blood cell count to 18 000/µL with neutrophilia.*<br><br>*Sputum cultures revealed H. Influenzae with current antibiogram (see projection).*<br><br>*Chest X-ray revealed a pneumonic infiltrate of the right upper lobe.*<br><br>*Faeces cultures revealed an ESBL-producing E. Coli.*<br><br>*After initial normalisation of temperature, CRP and white blood cell count, there has been a relapse of fever since yesterday.*
**R**ecommendation	● current antimicrobial policy<br>● own proposal<br>● further diagnostic approach<br><br>*Hemocultures and CSF cultures were taken. We are suspicious of a catheter infection or a central neurological infection. We would like to cover multiresistant gram negative bacteria.*

PART 2: Discussion with the ID ward round team (a senior pediatric intensive care consultant, a senior infectious disease consultant, a senior clinical pharmacist, a senior microbiologist). Formulation of recommendation.

PART 3: Wrap-up by the junior medical staff member.

Supplement 2 : Illustration COSARAPed

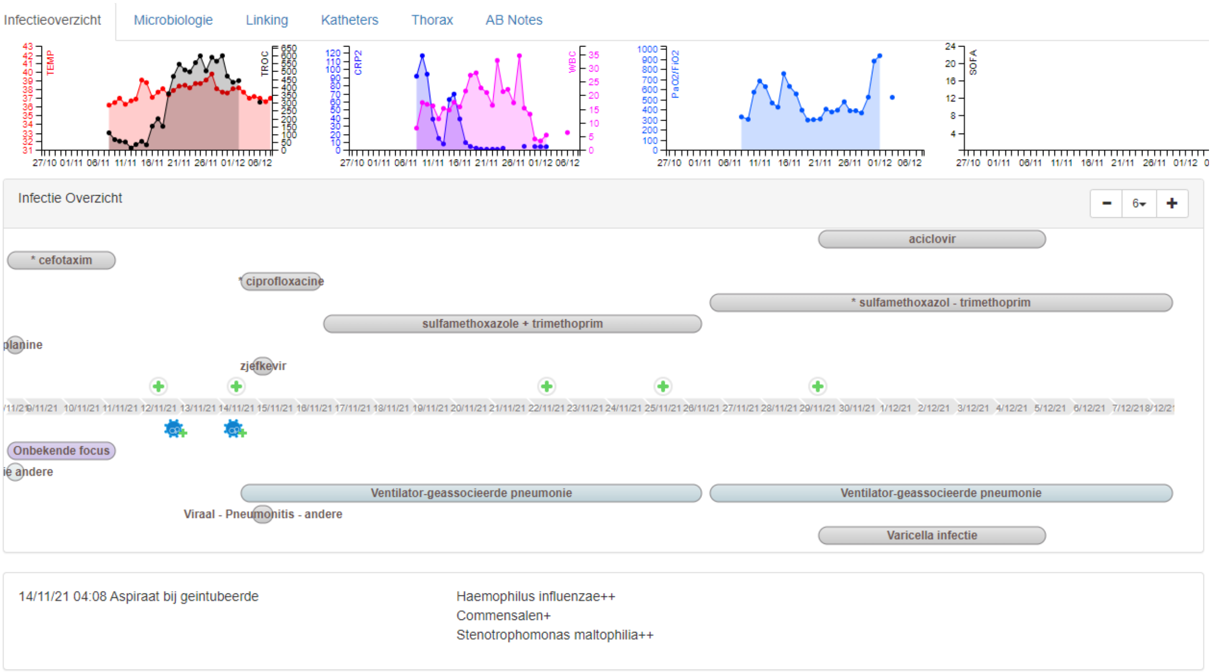

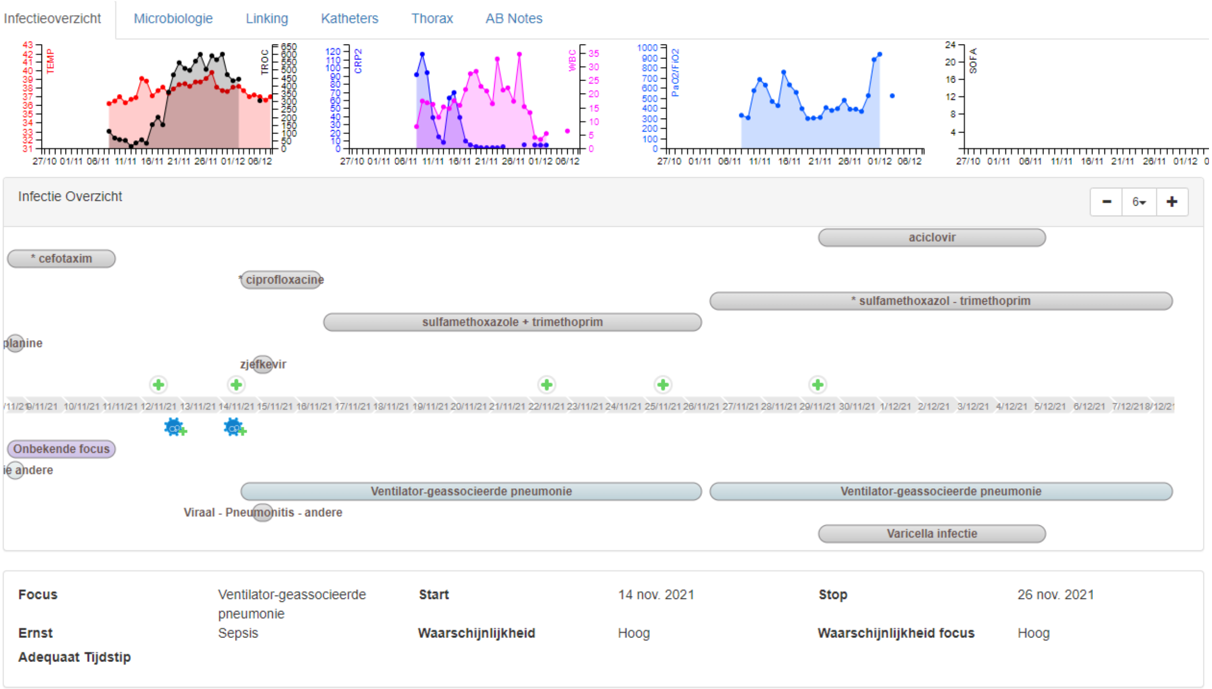

Infectieoverzicht | Microbiologie | Linking | Katheters | Thorax | AB Notes

Bacteriën | Stalen | Urine sediment

Tijdstip staalafname	Materiaal	Kulturen
29/11/21 12:21	Faeces/Anale wisser	1 Stenotrophomonas maltophilia .
29/11/21 12:21	Mond/Keeluitstrijk	1 Stenotrophomonas maltophilia .
25/11/21 10:07	Sputum	1 Commensalen ++<br>2 Stenotrophomonas maltophilia +
22/11/21 10:07	Mond/Keeluitstrijk	1 Stenotrophomonas maltophilia .
14/11/21 04:08	Aspiraat bij geintubeerde	1 Haemophilus influenzae ++<br>2 Commensalen +<br>3 Stenotrophomonas maltophilia ++
12/11/21 15:30	Aspiraat bij geintubeerde	1 Stenotrophomonas maltophilia ++<br>2 Commensalen +-

Lijst leegmaken

211114-1409

14/11/21 04:08

Materiaal: Aspiraat bij geintubeerde

Cultures: 1 Haemophilus influenzae ++

Amoxicilline	R
Amoxicilline - clavulaanzuur	R
Cotrimoxazol	S
Cefuroxim (IV)	R
Ofloxacine/levofloxacine	R

Infectieoverzicht | Microbiologie | Linking | Katheters | Thorax | AB Notes

Infectie	Medicatie	Verantwoordelijke culturen	Bijdragende culturen	Finaal
Varicella infectie<br>29 nov. 2021 - 5 dec. 2021	aciclovir<br>29 nov. 2021 - 5 dec. 2021	Empirisch		☑
Ventilator-geassocieerde pneumonie<br>26 nov. 2021 - 8 dec. 2021	sulfamethoxazol - trimethoprim<br>26 nov. 2021 - 8 dec. 2021	Stenotrophomonas maltophilia ++<br>14 nov. 2021 Aspiraat bij geintubeerde		☑
Viraal - Pneumonitis - andere<br>14 nov. 2021 - 15 nov. 2021	zjefkevir<br>14 nov. 2021 - 15 nov. 2021	Serologische diagnose		☑
Ventilator-geassocieerde pneumonie<br>14 nov. 2021 - 26 nov. 2021	sulfamethoxazole + trimethoprim<br>16 nov. 2021 - 26 nov. 2021	Stenotrophomonas maltophilia ++<br>14 nov. 2021 Aspiraat bij geintubeerde		☑
	ciprofloxacine<br>14 nov. 2021 - 16 nov. 2021	Stenotrophomonas maltophilia ++<br>12 nov. 2021 Aspiraat bij geintubeerde		☑
Onbekende focus<br>8 nov. 2021 - 11 nov. 2021	cefotaxim<br>8 nov. 2021 - 11 nov. 2021	Empirisch		☑
Profylaxie andere<br>8 nov. 2021 - 8 nov. 2021	teicoplanine<br>8 nov. 2021 - 8 nov. 2021	Empirisch		☑

Infectieoverzicht | Microbiologie | Linking | Katheters | Thorax | AB Notes

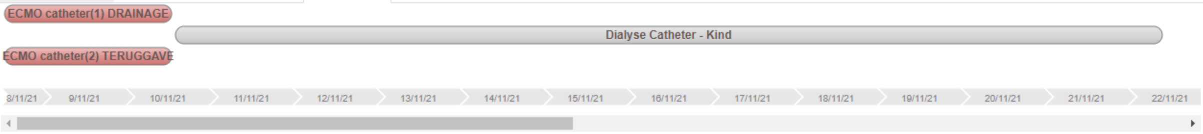

**Name**	ECMO catheter(1) DRAINAGE	**Starttime**	8/11/21 12:47	**Endtime**	10/11/21 13:51
**Place**	A carotis re	**Startlocatie**	Plaatsing		

Supplement 3 : Duration handover/discussion

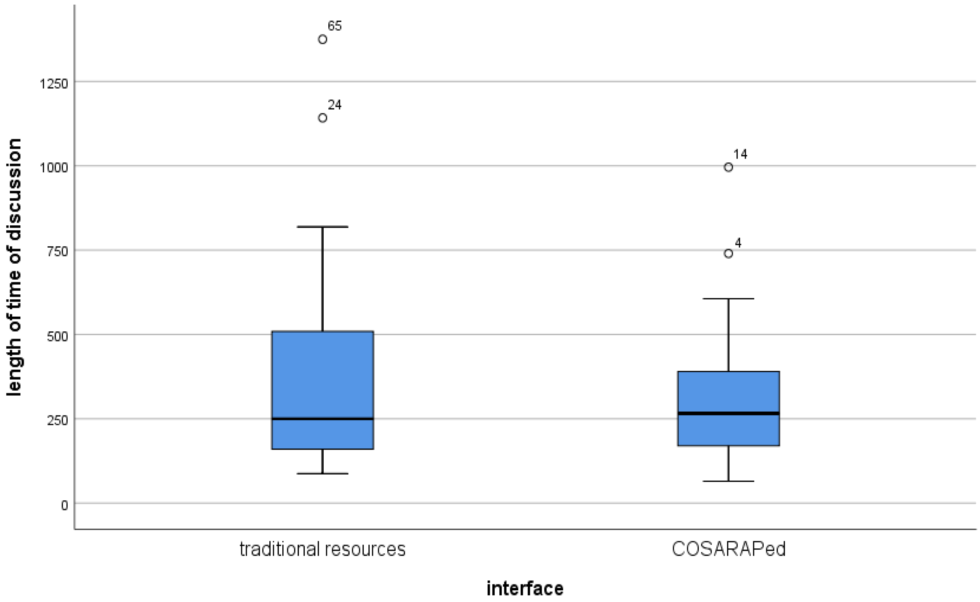

Supplement 4 : AS-interventions

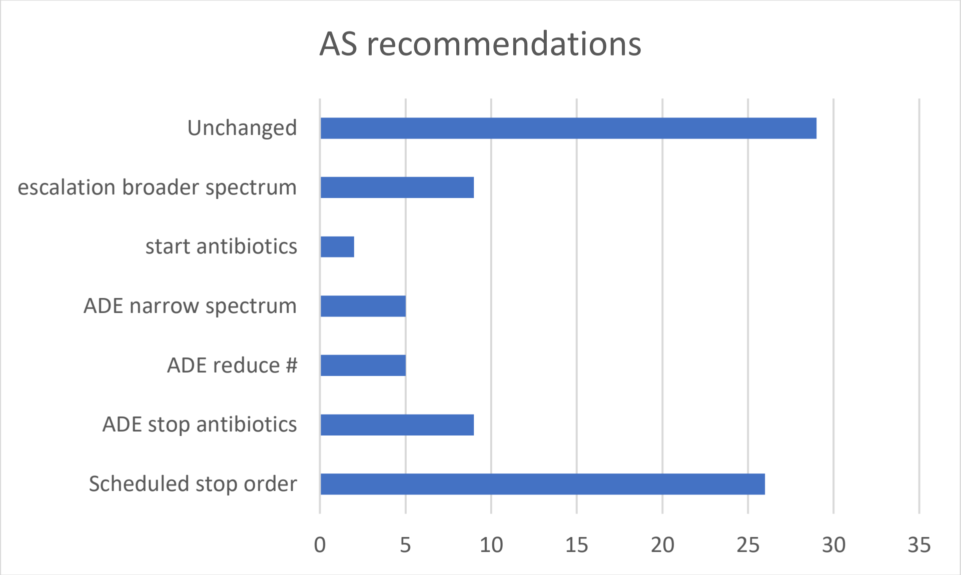

Graphic 2 : AS recommendations

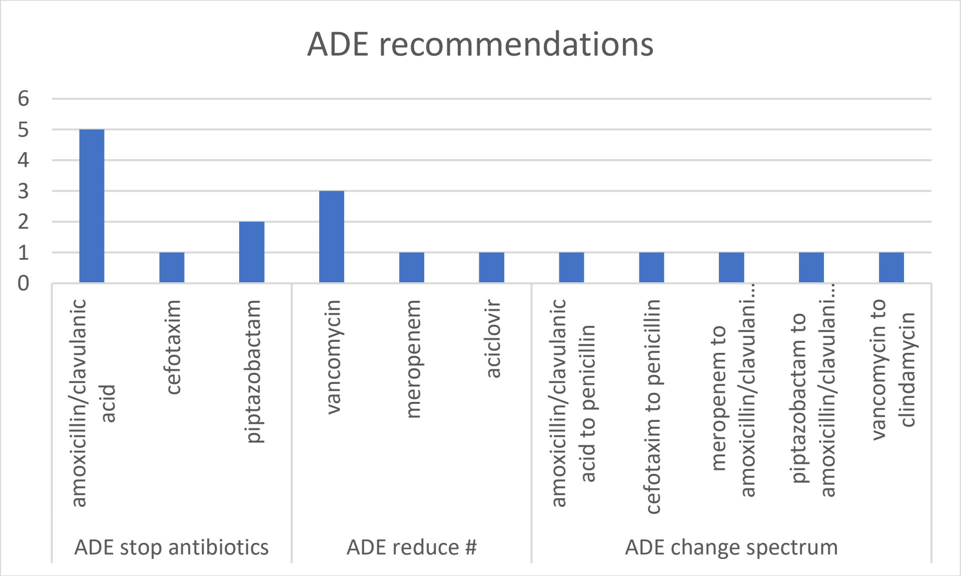

AS Interventions : ADE recommendations
